# Heartbeat of the Sun from Principal Component Analysis and prediction of solar activity on a millenium timescale

**DOI:** 10.1038/srep15689

**Published:** 2015-10-29

**Authors:** V. V. Zharkova, S. J. Shepherd, E. Popova, S. I. Zharkov

**Affiliations:** 1 Northumbria University, Department of Mathematics & Information Sciences, Newcastle upon Tyne, NE2 1XE, UK; 2 Institution of Space Science Research, Space Physics Department, Kiev, 03022, Ukraine; 3 University of Bradford, School of Engineering, Bradford, BD7 1DP, UK; 4 Skobeltsyn Institute of Nuclear Physics, Moscow 119234, Russia; 5 University of Hull, Department of Physics and Mathematics, Kingston upon Hull, HU6 7RX, UK

## Abstract

We derive two principal components (PCs) of temporal magnetic field variations over the solar cycles 21–24 from full disk magnetograms covering about 39% of data variance, with *σ* = 0.67. These PCs are attributed to two main magnetic waves travelling from the opposite hemispheres with close frequencies and increasing phase shift. Using symbolic regeression analysis we also derive mathematical formulae for these waves and calculate their summary curve which we show is linked to solar activity index. Extrapolation of the PCs backward for 800 years reveals the two 350-year grand cycles superimposed on 22 year-cycles with the features showing a remarkable resemblance to sunspot activity reported in the past including the Maunder and Dalton minimum. The summary curve calculated for the next millennium predicts further three grand cycles with the closest grand minimum occurring in the forthcoming cycles 26–27 with the two magnetic field waves separating into the opposite hemispheres leading to strongly reduced solar activity. These grand cycle variations are probed by *α* *−* Ω dynamo model with meridional circulation. Dynamo waves are found generated with close frequencies whose interaction leads to beating effects responsible for the grand cycles (350–400 years) superimposed on a standard 22 year cycle. This approach opens a new era in investigation and confident prediction of solar activity on a millenium timescale.

Solar activity is manifested in sunspot occurrence on the solar surface characterized by the smoothed sunspot numbers, which were selected as a proxy of solar activity (see, for example, the top plot in http://solarscience.msfc.nasa.gov/images/bfly.gif). The sunspot numbers show quasi-regular maxima and minima of solar activity changing approximately every 11 years, with changing leading magnetic polarity in a given hemisphere (or 22 years for sunspots with the same polarity) reflecting changing magnetic activity of the Sun^1^.

The longest direct observation of solar activity is the 400-year sunspot-number series, which depicts a dramatic contrast between the almost spotless Maunder and Dalton minima, and the period of very high activity in the most recent 5 cycles^2^,^3^, prior to cycle 24. Many observations indicate essential differences between the activity occurring in the opposite hemispheres for sunspots^4^ and for solar and heliospheric magnetic fields^5^.

Prediction of a solar cycle through sunspot numbers has been used for decades as a way of testing accuracy of solar dynamo models, including processes providing production, transport and disintegration of the solar magnetic field. Cycles of magnetic activity are associated with the action of a dipole solar dynamo mechanism called ‘*α* − Ω dynamo’^6^. It assums the action of solar dynamo to occur in a single spherical shell, where twisting of the magnetic field lines (*α*-effect) and the magnetic field line stretching and wrapping around different parts of the Sun, owing to its differential rotation (Ω-effect), are acting together^7^,^8^.

As a result, magnetic flux tubes (toroidal magnetic field) seen as sunspots are produced from the solar background magnetic field (SBMF) (poloidal magnetic field) by a joint action of differential rotation (Ω-effect) and radial shear (*α*-effect), while the conversion of toroidal magnetic field into poloidal field is governed by the convection in the rotating body of the Sun. The action of the Coriolis force on the expanding, rising (compressed, sinking) vortices results in a predominance of right-handed vortices in the Northern hemisphere and left-handed vortices in the Southern hemisphere leading to the equatorward migration of sunspots during a solar cycle duration visible as butterfly diagams (see http://solarscience.msfc.nasa.gov/images/bfly.gif, the bottom plot).

The last few decades were extremely fruitful in investigating the contribution of various mechanisms to the dynamo processes including the conditions for dynamo wave generation from the mean dynamo models with different properties of solar and stellar plasmas, as discussed in the recent reviews^7^,^8^.

As usual, the understanding of solar activity is tested by the accuracy of its prediction. The records show that solar activity in the current cycle 24 is much lower than in the previous three cycles 21–23 revealing more than a two-year minimum period between cycles 23 and 24. This reduced activity in cycle 24 was very surprising because the previous five cycles were extremely active and sunspot productive forming the Modern Maximum^2^,^3^. Although the reduction of solar activity in cycle 24 led some authors to suggest that the Sun is on its way towards the Maunder Minimum of activity^9^.

However, most predictions of solar activity by various methods, such as considering linear regression analysis^10^, neural network forecast^11^, or a modified flux-transport dynamo model calibrated with historical sunspot data from the middle-to-equator latitudes^12^, anticipated a much stronger cycle 24^10^. There were only a few predictions of the weaker cycle 24^13^ obtained with the high diffusivity Babcock-Leighton dynamo model applied to polar magnetic fields as a new proxy of solar activity. However, a dynamo model with a single wave was shown to be unable to produce reliable prediction of solar activity for longer than one solar cycle because of the short memory of the mean dynamo^14^.

Consistent disagreement between the sunspot numbers, measured averaged sunpost numbers and the predicted ones by a large number of complex mathematical models for cycle 24, is undoubtedly the result, which emphasizes the importance of different physical processes occurring in solar dynamo and affecting complex observational appearance of sunspots on the surface.

## Results

### Two principal components as two dynamo waves

In order to reduce dimensionality of these processes in observational data, Principal Component Analysis (PCA) was applied^15^ to low-resolution full disk magnetograms captured by the Wilcox Solar Observatory^16^. This approach revealed a set of more than 8 independent components (ICs), which seem to appear in pairs^15^, with two principal components (PCs) covering about 39% of the variance of the whole magnetic field data, or standard deviation of *σ* = 0.67. The main pair of PCs is associated with two magnetic waves of opposite polarities attributed to the poloidal field produced by solar dynamo from a dipole source^17^.

**Figure 1:**
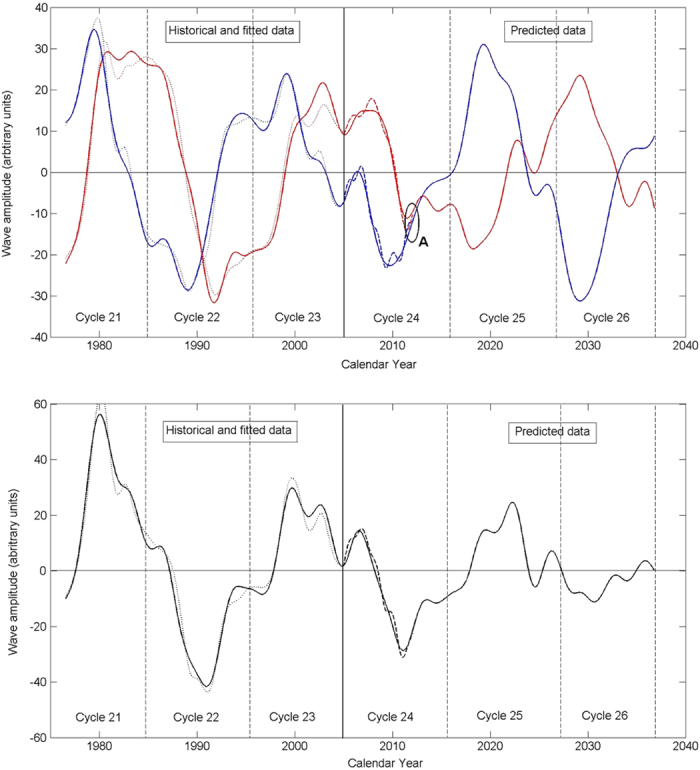
Top plot: the two principal components (PCs) of SBMF (blue and red curves) obtained for cycles 21–23 (historic data^15^) and predicted^19^ for cycles 24–26 with the Eqs. (2)–(3)(2)–(3). The dotted lines show the PCs derived from the data and the solid lines present the curves plotted from formulae 2 (blue) and 3 (red). The accuracy of fit of the both PC curves is better than 97%. The point A shows the current time. The cycle lengths (about 11 years) are marked at the minima by the vertical lines. The bottom plot: The summary PC derived from the two PCs above for the ‘historical’ (21–23) and predicted cycles (24–26) data. The dotted curve shows PCs derived from the data and the solid line - from the the solid curves from the top plot using formulae 2–3. The cycle lengths (about 11 years) are again marked by the vertical lines at the cycle minima. All the plots are a courtesy of Shepherd *et al.*^19^. © AAS. Reproduced with permission.

The two principal components (PCs) derived from solar background magnetic field (SBMF)^15^ (cycle 21–23) and predicted for cycle 24–26 are presented in Fig. 1 (the upper plot). For the first time PCA allowed us to detect, *two magnetic waves* in the SBMF^15^ and not a single one assumed in the mean dynamo models. These waves are found originating in the opposite hemispheres and travelling with an increasing phase shift to the Northern hemisphere in odd cycles and the Southern hemisphere in even cycles^15^. This can explain the well-observed North-South asymmetry in sunspot numbers, background magnetic field, flare occurences and so on (see Zharkov *et al.*^4^ and references therein) defining the active hemisphere for odd (North) and even (South) cycles.

**Figure 2:**
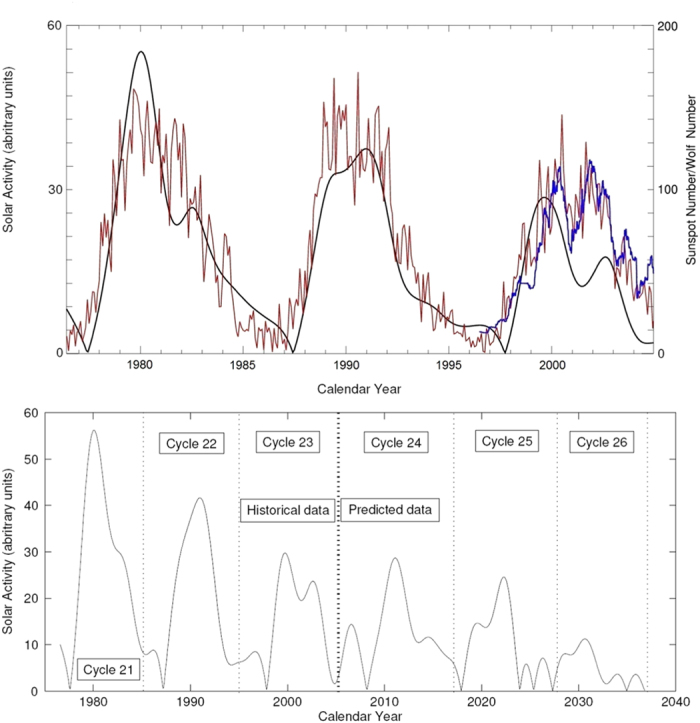
Top plot: Comparison of the modulus summary curve (black curve) obtained from the summary curve inFig. 1 with averaged sunspot numbers (brown curve) and magnetic fiel (blue curve) for cycles 21–23. Bottom plot: The modulus summary curve associated with the sunspot numbers derived for cycles 21–23 (plotted in the top plot) and calculated for cycles 24–26 using the mathematical formulae (2–3). The plots are a courtesy of Shepherd *et al.*^19^. © AAS. Reproduced with permission.

The formation of magnetic flux tubes emerging on the solar surface as sunspots can be considered as a result of interaction in the solar interior of the two magnetic waves of the solar background magnetic field^15^ when their phase shift is not very large. These two magnetic waves of the poloidal field can account for the observed sunspot magnetic field^18^, or averaged sunspot numbers, after their amplitudes are added together into the summary wave (Fig. 1, bottom plot) and converted to the modulus curve by taking modulus of the summary curve^19^ (Fig. 2, bottom plot). The modulus curve plotted for cycles 21–23 in Fig. 2 (top plot) corresponds rather closely to the averaged sunspot numbers for cycles 21 and 22 while being noticeably lower than the sunspot curve for cycle 23, which anticipated the recently discovered sunspot calibration errors occurred in the past few decades^20^.

The maximum (or double maximum for the waves with a larger phase shift of solar activity for a given cycle) coincides with the time when each of the waves approaches a maximum amplitude and the hemisphere where it happens becomes the most active one. This can account naturally for the north-south asymmetry of solar activity often reported in many cycles. Also the existence of two waves in the poloidal magnetic field instead of a single one, used in most prediction models, and the presence of a variable phase difference between the waves can naturally explain the difficulties in predicting sunspot activity on a scale longer than one solar cycle with a single dynamo wave^14^ since the sunspot activity is associated with the modulus summary curve of the two dynamo waves^19^ that is a derivative from these two waves.

### Mathematical description of the observed magnetic waves

Amplitude and frequency variations of these waves, or PCs, over time are found using symbolic regression analysis^21^ with Euriqa software (see the Methods section for data analysis^19^). The wave amplitudes follow the product of two cosine functions (cos * cos), while the frequencies folow a nested function (cos (cos)) depicting the fact that the waves periodically change their frequency and phase with time. These formulae are used to extract the key parameters of the principal components of SBMF waves, which are, in turn, used for prediction of the overall level of solar activity for solar cycles 24–26 associated with the averaged sunspot numbers^19^. The accuracy of these formulae for prediction of the principal components is tested for cycle 24 showing the predicted curve fitting very closely (with an accuracy of about 97.5%) the PCs derived from the observations of SBMF and sunspot numbers^19^.

For the forthcoming cycles 25 and 26 (Fig. 1) the two waves are found to travel between the hemispheres with decreasing amplitudes and increasing phase shift approaching nearly a half period in cycle 26. This leads, in fact, to a full separation of these waves in cycle 26 into the opposite hemispheres^19^. This separation reduces any possibility for wave interaction for this cycle that will result in significantly reduced amplitudes of the summary curve and, thus, in the strongly reduced solar activity in cycle 26^19^, or the next Maunder Minimum^9^ lasting in 3 cycles 25–27.

### Prediction of solar activity on millennium scale

**Figure 3:**
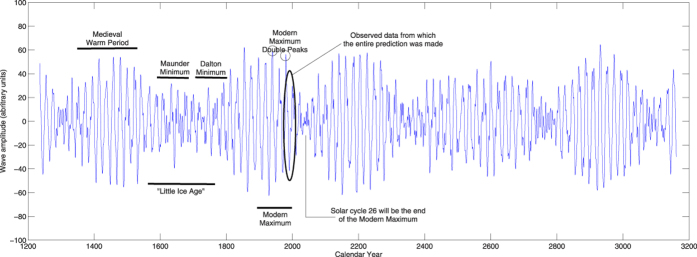
The predicted summary wave (the sum of two principal components) calculated from 1200 to 3200 years from the ‘historical’ period (cycles 21–23) marked with a black oval. The historical maxima and minima of the solar activity in the past are marked by the horizontal brackets.

By far the most impressive achievement to-date of this approach is its ability to make very long term predictions of solar activity with high accuracy over the timescales of many centuries. The summary curve of the two principal components (magnetic waves) expressed by the formulae (2 and 3) in the Method of data analysis^19^ is calculated backwards and forwards for the period 1200–3200 years as shown in Fig. 3.

Remarkably, our current prediction of the summary curve backwards by 800 years shown in the left (from oval) part of Fig. 3, corresponds very closely to the sunspot data observed in the past 400 years as indicated by the brackets in Fig. 3, with the black oval marking the data used to derive Eq. (2) and (3) defining the wave variations. We predict correctly many features from the past, such as: 1) an increase in solar activity during the Medieval Warm period; 2) a clear decrease in the activity during the Little Ice Age, the Maunder Minimum and the Dalton Minimum; 3) an increase in solar activity during a modern maximum in 20th century.

This visual correspondence in the features between the summary curve and the averaged sunspot numbers is most surprising, given the fact that the principal components are derived from the solar background magnetic field, and they are not linked directly to the sunspot numbers (see Methods for data analysis) besides the modulus summary curve derived from the principal components as shown in Fig. 2.

The summary curve reveals a superposition of the amplitudes of the two dynamo waves, or a ‘beating’ effect creating two resulting waves: one of higher frequency (corresponding to a classic 22-year cycle) and a second wave of lower frequency (corresponding to a period of about 350–400 years), which modulates the amplitude of the first wave. It appears that this grand cycle has a variable length from 320 years (in 18–20 centuries) to 400 (in 2300–2700) predicted for the next millennium. Amplitudes in the shorter grand cycles are much higher than the amplitudes in the longer ones.

This long-term ‘grand’ cycle was previously postulated in 1876 by Clough^22^ as a 300-year cycle superimposed on the 22 year cycle using the observations of aurorae, periods of grape harvests etc, which was later suggested to have a period of about 205 years^23^. These periods are close to those reported for the last 800 years in the summary curve plotted in Fig. 3 derived from the observed magnetic field variations.

The spectacular accuracy of the historical fit in the past 800 years gave us the confidence to extrapolate the data into the future for a similar epoch of 1200 years (Fig. 3, right part of the curve) clearly showing, as expected, several 350–400-year grand cycles. We note, in particular, a decreasing activity for solar cycles 25 and 26 coinciding with the end of the previous 350–400-year grand cycle and then increase of the solar activity again from cycle 27 onwards as the start of a new grand cycle with an unusually weak cycle 30. Hence, cycles 25–27 marks a clear end of the modern grand period that can have significant implications for many aspects of solar activity in human lives including the current debate on climate change.

## Discussion

### Preliminary interpretation with the two layer *α* − Ω dynamo model

**Figure 4:**
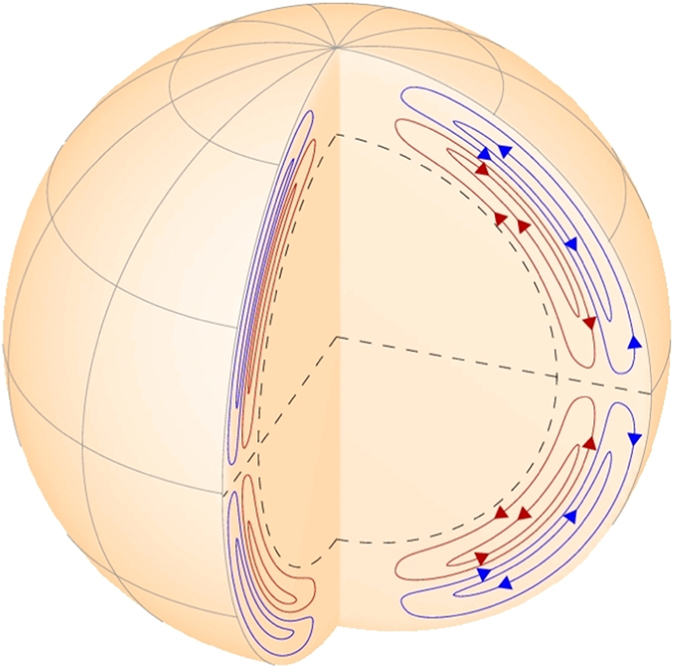
The schematic dynamo model with two cells in the solar interior having the opposite meridional circulation as derived from HMI/SDO observations by Zhao *et al.*^24^. © AAS. Reproduced with permission.

Now let us attempt some preliminary interpretation of the two principal components, or two magnetic waves of solar poloidal field, generated by the solar dynamo in two different cells, similar to those derived by Zhao *et al.*^24^ from helioseismological observations (Fig. 4), in order to fit the background magnetic field observations (Figs 1 and 3). This can be achieved with the modified Parker’s non-linear two layers dynamo model for two dipoles^17^ with meridional circulation: in the layer 1 of the top cell and layer 2 of the bottom cell from Fig. 4 (see Methods section for the model description) tested for the interpretation of latitudinal waves in the solar background magnetic field for cycles 21–23^17^ derived with PCA^15^.

**Figure 5:**
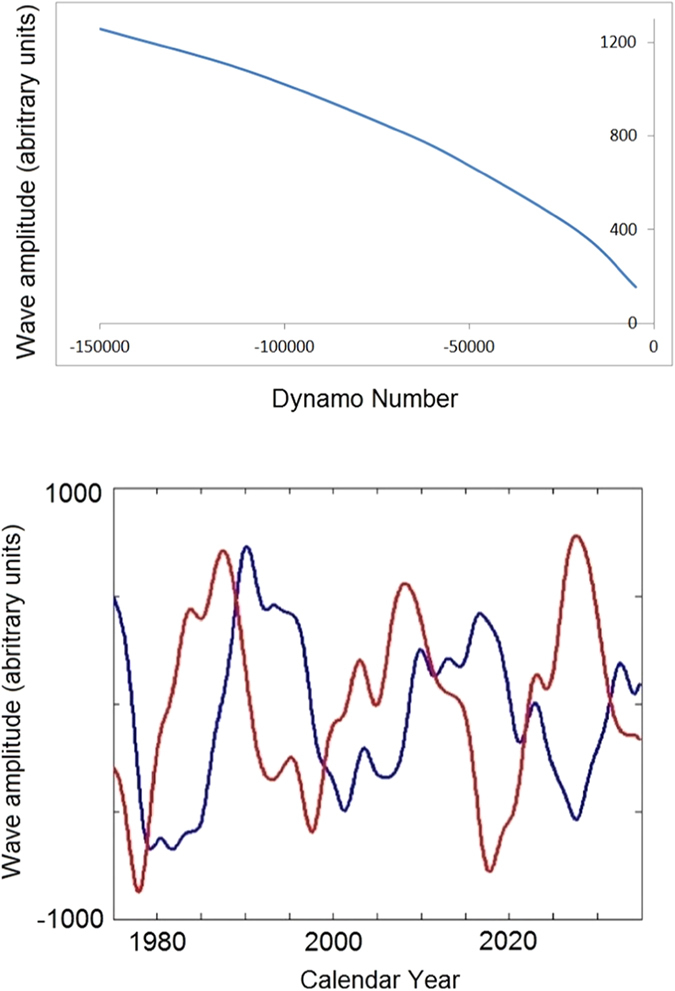
Top plot: Dependence of the solar dynamo-number *D* = *R*_*α*_*R*_Ω_ on a magnitude of the toroidal magnetic field (for detials of the parameters see the text). Bottom plot: Variations of the toroidal magnetic field simulated for cycles 21–26 with two layer *α*Ω dynamo model (see Methods section) for the inner (red line) and upper (blue line) layers. One arbitrary unit corresponds to 1–1.5 Gauss (see text for details).

The simulation results presenting the toroidal magnetic field are plotted in Fig. 5 (bottom plot) derived from the poloidal field (Fig. 1, top plot) for a period of six 11-year cycles using the dynamo equations (16–19) from Popova *et al.*^17^. The curves for poloidal (derived with PCA) and toroidal fields (simulated with the dynamo model) are found to have similar periods of oscillations whilst having opposite polarities (or having the phase shift of a half of the period), being in anti-phase every 11 years as previously reported^4^,^25^. The amplitude of generated toroidal magnetic field is plotted versus the dynamo number 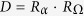 in Fig. 5 (top plot).

Furthermore, in cycles 25–27 and, especially, in cycle 26, the toroidal magnetic field waves generated in these two layers become fully separated into the opposite hemispheres, similar to the two PC waves attributed to poloidal field (Fig. 1, top plot), that makes their interaction minimal. This will significantly reduce the occurance of sunspots in any hemisphere, that will result in a very small solar activity index for this cycle, resembling the Maunder Minimum occurred in the 17th century.

**Figure 6:**
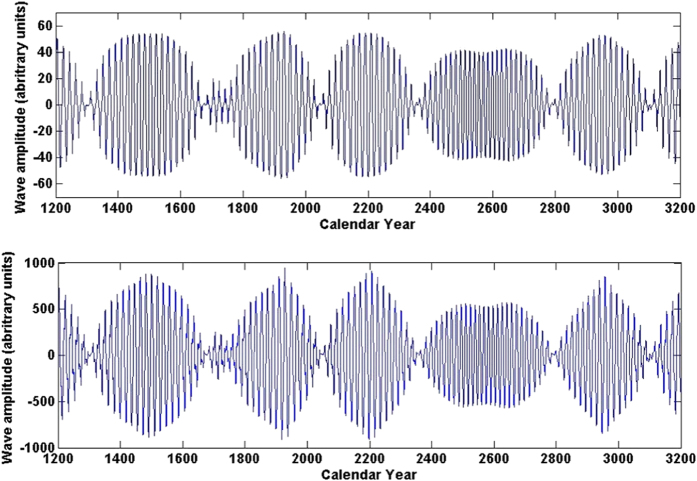
Variations of the summary poloidal (top plot) and toroidal (bottom plot) magnetic fields simulated for 2000 years with the two layer *α*Ω-dynamo model (see Methods section) with the parameters derived from the two PCs fromFig. 1 using mathematical formulae (2–3). One arbitrary unit corresponds to 1–1.5 Gauss (see text for details).

Using the same dynamo parameters derived from the observed principal components for these 6 cycles, let us extend the calculation (see the Methods for details) to a longer period of two millennia shown in Fig. 6 for both poloidal (top plot) and toroidal (bottom plot) fields. According to the dynamo theory and analysis of observational data^7^,^27^ the generated toroidal field is much stronger than the poloidal. Although, exact values of the amplitudes of these fields in the solar convection zone are unknown and estimated from dynamo models. In our simple model the amplitude of toroidal field at the maximum is about 1000 Gauss, and of the poloidal one is of the order of several tens of Gauss. Hence, in Figs 5 and 6 one arbitrary unit approximately corresponds to 1–1.5 Gauss.

It can be seen that variations of the model magnetic fields (Fig. 6) generated by the two dipole sources located in diferent layers reproduce the main features discovered in Fig. 3, e.g modulation of the amplitude of 22 year cycle by much slower oscillations of about 350 years, different duration (320–400) and amplitudes of different grand cycles. These variations are governed by different dynamo parameters as discussed below.

### Beating effect of two dynamo waves with close frequencies

The waves generated by a dynamo mechanism in each layer are found to have similar (but not equal) frequencies caused by a difference in the meridional flow amplitudes in the two layers (Fig. 5, bottom plot). In order to reproduce the summary curve in Fig. 3 from the two original waves, or PCs, the dynamo waves generated in different layers with an amplitude *A*_0_ have to have close but not equal frequencies *ω*_1_ and *ω*_2_ (or periods varying between 20 and 24 years), similar to Gleissberg’s cycle^7^,^26^.

The interference of these waves enabled by diffusion of the waves in the solar interior from the bottom to the top layer^27^ leads to formation of the resulting envelope of waves *Y*(*t*), or beating effect (see Fig. 3 and theoretical plots in Fig. 6), showing oscillations of a higher frequency 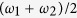 within the envelope and those of the envelope itself with a lower frequency of 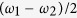 (or in *a grand cycle*) as follows:


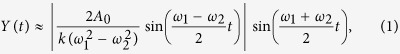


where *k* is some parameter defining properties of the solar interior where the waves propagate, e.g. diffusivity, dynamo number (*α* and Ω effects) and meridional circulation.

### Frequency and period variations

The beating effect between these frequencies can easily explain seemingly sporadic variations of high frequency amplitudes and the period of the low-frequency envelope wave in the resulting grand cycles seen in both the observational curve (Fig. 3 and theoretical curves (Fig. 6) reproducing the observational one. The higher the difference of frequencies the larger is the frequency, or a shorter period, of the grand cycle (350 years) and the smaller is a number of high frequency waves (≈22 year period) within this grand cycle. This effect is clearly seen in Figs 3 and 6, where the grand periods with a lower number of 22 year cycles are shorter (300–340 years, 2nd, 3rd and 5th grand cycles in Fig. 3), while those with higher number of 22-year cycles are longer (360–400 years, the 1st and 4th in Fig. 3).

The difference in frequencies of the dynamo waves in two layers is governed by the variations of velocities of meridional circulations in the very top and the very bottom zones of these two layers (see the Method section) (schematically presented in Fig. 4 from Zhao *et al.*^24^). The frequency of a wave is reduced (or its period is increased) when the meridional circulation has higher velocities and this frequency is increased (or its period is decreased) when the meridional circulation is slower. It means that the meridional circulation acts as a drag force for dynamo waves generated in each layer altering their natural frequencies that would occur without the circulation.

For example, within the two layers model considered, and taking into account that the low frequency cycles can have length *T*_*g*_ from 20 to 24 years (variations within Gleissberg’s cycle^7^), in order to produce the grand cycle with a beating period of 350 years, the periods of the dynamo waves in two layers should vary as follows: for the sunspot activity period *T*_*g*_ = 20 years -for the inner layer wave 1 − *T*_1_ = 18.9 years (corresponding to the velocity of meridional circulation about V = 7–8 m/s), for the upper layer wave 2 − *T*_2_ = 21 years (V = 9–10 m/s); for the activity period *T*_*g*_ = 24 years: the inner layer wave 1 − *T*_1_ = 22.46 years (V = 10–11 m/s), the upper layer wave 2 − *T*_2_ = 25.8 years (V = 13–14 m/s).

If the grand cycle is 400 years, then the dynamo wave periods in two layers would slightly change; e.g. for the cycle period *T*_*g*_ = 20 years - for the inner layer wave 1 −*T*_1_ = 19 years (V = 7–8 m/s), for the upper layer wave 2 − *T*_2_ = 21 years (V = 9–10 m/s); for the period of *T*_*g*_ = 24 years: the inner layer wave 1 − *T*_1_ = 22.6 years (V = 10–11 m/s), the upper layer wave 2 − *T*_2_ = 25.53 years (V = 13–14 m/s).

It can be seen that the period of the wave 1 generated in the inner layer (at the bottom of the convective zone) remains more or less stable at about *T*_1_ = 19 years (for generation of the low frequency activity period *T*_*g*_ = 20 years) or *T*_1_ = 22.6 year (for *T*_*g*_ = 24 years). While the period of the wave 2 generated in the upper layer should have larger fluctuations (e.g. *T*_2_ = 25.8 years for 350 grand cycle versus *T*_2_ = 25.53 years for 400 years grand cycle). These fluctutation are likely to be affected by the physical conditions in the solar interior, where the wave 2 is formed and the wave 1 has to travel through and to interact with the wave 2 to cause the beating effect combining the grand (ranging in 300–400 years) and short (ranging in 20–24 years) cycles seen in Fig. 3 as reproduced with the dynamo model in Fig. 6 for both poloidal and toroidal magnetic fields.

Of course, estimations of the wave beating above are rather preliminary, given the fact that the PCs (or dynamo waves) in each layers comprise at least 5 waves with close frequencies as discussed in the Method section (Eqs. 2 and 3). This results in much more complex beating effects derived from PCA as presented in Fig. 3. The dynamo calculations only partially reproduced the long cycle with a period of about 350 years, which is the same for the whole millennium. However, in order to reproduce the full summary curve with the variable long-term period in Fig. 3 more detailed dynamo simulations including quadruple magnetic sources in all the three layers (shown in Fig. 4) are required.

### Wave amplitude variations

The amplitudes of dynamo waves are affected by the variations of both *α* and Ω effects, or by the dynamo number *D*, i.e. a decrease of the negative dynamo number *D* (or its increase in absolute value) leads to an increase of toroidal field amplitude (see Fig. 5, top plot).

This effect can be observed in both the observational (Fig. 3) and theoretical (Fig. 6) plots. In shorter grand cycles (with periods of 300–340 years), e.g. in 1800–2000 years and 2100–2350 years, the amplitudes of the high frequency wave (*T*_*g*_ = 20–24 years) are much higher than in longer cycles (periods of 350–400 years) in 1300–1650 years or 2400–2800 years. Although, in order to reproduce more closely the whole variety of observational features on a longer timescale, more detailed 3D model simulations are required.

Therefore, the derived mathematical laws in cyclic variations of principal components of the observed solar magnetic field, which fit closely most of the observational features of solar activity in the past as shown in Fig. 3 and reproduced by the dynamo model in Fig. 6 opens a new era in the investigation of solar activity on millennium scale. By combining the observational curve with simulations of solar dynamo waves in two layers, it is possible to derive better understanding of the processes governing solar activity and produce long-term prediction of solar activity with impressive accuracy.

## Methods

### Derivation of parameters of the observed magnetic waves

In order to distill the main parameters of the waves present in the observational solar magnetic data, one needs to reduce their dimensionality with the Principal Component Analysis (PCA)^28^. PCA is an orthogonal linear transformation allowing a vector space to be transformed to a new coordinate system, reducing the multi-dimensional data to lower dimensions for analysis, so that the greatest variance by any projection of the data lies on the first coordinate called the Principal Component (PC) with the second PC orthogonal to the first is defined by the second largest variance. This technique simultaneously (i) reduces the data dimensionality, (ii) increases the signal-to-noise ratios and (iii) orthogonalises the resulting components so that they can be ascribed to separate physical processes (see Zharkova *et al.*^15^ for more details). The PCA is an exact method, and its accuracy defined only by the noise of measurements, 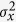, of the original vector^29^.

PCA was applied to low-resolution full disk solar background magnetic field (associated with the poloidal magnetic field) only become available from cycle 21 to cycle 24 as measured by the Wilcox Solar Observatory (with accuracy better than 0.5 Gauss, or the measurement error 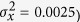. We derive the dominant eigenvalues (0.1 and 1.0) covering the maximum variance of 39%^15^ defining the eigenfunctions, or Principal Components (PCs), which came as a pair of waves. These PCs are considered as the main (dipole) dynamo waves of the solar poloidal magnetic field.

By applying a 3-year running averaging filter, any short-term (<3 years) fluctuations of magnetic field data are removed allowing us to keep the accuracy of PCA not worse than the measurement error (Wentzell and Lohnes^30^). The overall PCA accuracy of defining its eigen values from the WSO data with known measurement error (see Faber *et al.*^29^) is not worse than 0.2%. Running PCA on a combination of magnetic field measurements for any two cycles, or for all four cycles21–24 produces, within the error of 0.2%, the same eigenvalues as for the three cycles used in PCA^15^.

For classification of the derived PCs we apply the symbolic regression approach based on the Hamiltonian principle implemented in *the Euriqa* software^21^. This allows us to derive the exact mathematical formulae for the amplitude variations and phase shifts of both principal components as follows^19^:

for wave 1:


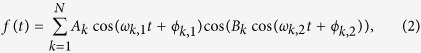


for wave 2:


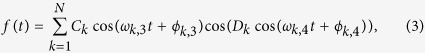


where the parameters with *ω* define the corresponding wave frequencies and *ϕ* define their phase shifts. Shepherd *et al.*^19^ found that the approximations with only N = 5 terms in the series above allow them to capture the functions describing the waves of PCs for the cycles 21–24 with an accuracy better than 97%^19^. As expected, any attempts to distill the parameters from the original magnetic field data (before deriving PCs) were unsuccessful indicating the very complex nature of the original magnetic field waves.

These two PCs are used for calculation of the summary wave (a sum of amplitudes) and the modulus summary wave (reflected to the positive amplitudes only) linked to the averaged sunspot numbers currently used for definition of solar activity.

### Non-linear *α*Ω dynamo model in a two-layer medium with meridional circulation

In order to understand the basic features of the derived PCs, let us use Parker’s *α*Ω-dynamo model with two layers with meridional circulation^17^ updated by considering a non-linear dynamo process. It is assumed that dynamo waves are generated by the dipole sources only located in two layers: one dipole in the subsurface layer and the other dipole deeply in the solar convection zone (see Fig. 4); and the parameters (dynamo number and meridional circulation) of magnetic field generation in each layer are different^17^.

This results in the simultaneous existence of two magnetic waves with different periods and phase shifts^17^, similar to those derived with PCA (see Fig. 1). For the sake of simplicity this approach excludes the dynamo waves generated by quadruple sources in both layers accounting for the other six independent components^17^, which are shown to slightly modify the overall appearance of magnetic waves that will be considered in the forthcoming paper.

The dynamo equations describing the generation and evolution of the solar magnetic field in a two-layer medium, are obtained from a system of electrodynamic equations for mean fields in the assumption that the dynamo wave propagates in a thin spherical shell^17^. In this case, the magnetic field is averaged along the radius within a certain spherical shell and the terms describing the curvature effects near the pole are excluded. In addition, in this approximation, we assume that the magnetic field is generated independently in either layer. As a result, the equations take the form of equations (16–19) by Popova *et al.*^17^ solved numerically using the method of lines^31^ and verified analytically by the low-mode approach^32^.

In these equations the dynamo number 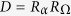 is defined by the parameters *R*_*α*_ and *R*_Ω_ describing, respectively, the intensity of *α*-effect, and the differential rotation, or Ω-effect. The latitudinal profile of the poloidal magnetic field is assumed for simplicity to be proportional cos(*θ*) where *θ* is the solar latitude measured from the equator.

We consider the *α*-effect the amplitude F(t, coordinates) and widely used algebraic quenching^7^ in a form:


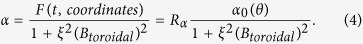


For calculations the amplitude of *α* effect, *R*_*α*_ is moved to the dynamo number *D*, while the algebraic quenching of the helicity is used for stabilization of a magnetic field growth, e.g. redefining 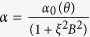, where 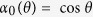 is the helicity in unmagnetized medium and 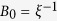 is the magnetic field, for which the 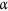-effect is considerably suppressed.

The contribution of differential rotation into the generation of magnetic field is defined^27^ by the terms 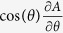 for one layer or 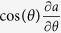 for another layer, following the general trend of being maximum at the equator and minimal at the poles. The dynamo number 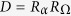 also includes an amplitude of differential rotation *R*_Ω_, which can vary in different layers.

Since the frequencies of magnetic waves generated by dynamo mechanism are known to be mostly affected by meridional circulation velocities^17^,^32^ while their amplitudes are governed by the variations of dynamo number *D*, then the PC waves can be reproduced in different layers with different dynamo numbers and slightly different meridional circulation velocities.

In each layer we consider a 1D dynamo model with meridional circulation *V* being a function dependent only on *θ* e.g. 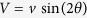, so that it vanishes at the poles and is maximal at the middle latitudes approaching amplitudes of 9–15 m/s^24^,^33^. Also, to comply with the material conservation rule, the meridional circulation the multi-cellular meridional circulation has to have the opposite directions in upper and inner layers of the cells in as shown in Fig. 4 suggested earlier by Dikpati^34^,^35^ and Popova *et al.*^17^ that was recently confirmed from the helioseismic observations with HMI by Zhao *et al.*^24^.

In general, there are three layers (see Fig. 4), in which the meridional circulation affects the magnetic field: 1- the very top layer in the upper cell and 2- the very bottom layer in the inner cell where the meridional circulation has the same direction but different velocities and 3- the middle layer where cells have a boundary and their the circulation has the opposite direction, complying with the mass conservation law. In the current model we consider the top and bottom layers (1–2) only to reproduce the long-term oscillations produced in them, while oscillations in the middle layer 3 affecting the short-term biennial^32^ are out of scope of the current study.

For each layer the principal components (PCs) of poloidal magnetic field were substituted into the dynamo equations (17 and 19) of Popova *et al.*^17^, from which the corresponding toroidal magnetic field components are derived fitting these PCs. Then we substitute the toroidal and poloidal components into the dynamo equations (16 and 18) of Popova *et al.*^17^ for corresponding layers and derive their dynamo numbers *D*.

## Additional Information

**How to cite this article**: Zharkova, V. V. *et al.* Heartbeat of the Sun from Principal Component Analysis and prediction of solar activity on a millennium timescale. *Sci. Rep.* **5**, 15689; doi: 10.1038/srep15689 (2015).
